# Application of Machine Learning in a Rodent Malaria Model for Rapid, Accurate, and Consistent Parasite Counts

**DOI:** 10.4269/ajtmh.24-0135

**Published:** 2024-09-10

**Authors:** Sean Yanik, Hang Yu, Nattawat Chaiyawong, Opeoluwa Adewale-Fasoro, Luciana Ribeiro Dinis, Ravi Kumar Narayanasamy, Elizabeth C. Lee, Ariel Lubonja, Bowen Li, Stefan Jaeger, Prakash Srinivasan

**Affiliations:** ^1^ Department of Molecular Microbiology and Immunology, Johns Hopkins School of Public Health, Baltimore, Maryland;; ^2^ Malaria Research Institute, Johns Hopkins School of Public Health, Baltimore, Maryland;; ^3^ National Library of Medicine, National Institutes of Health, Bethesda, Maryland;; ^4^ Department of Computer Science, Johns Hopkins Whiting School of Engineering, Baltimore, Maryland

## Abstract

Rodent malaria models serve as important preclinical antimalarial and vaccine testing tools. Evaluating treatment outcomes in these models often requires manually counting parasite-infected red blood cells (iRBCs), a time-consuming process, which can be inconsistent between individuals and laboratories. We have developed an easy-to-use machine learning (ML)-based software, Malaria Screener R, to expedite and standardize such studies by automating the counting of *Plasmodium* iRBCs in rodents. This software can process Giemsa-stained blood smear images captured by any camera-equipped microscope. It features an intuitive graphical user interface that facilitates image processing and visualization of the results. The software has been developed as a desktop application that processes images on standard Windows and MacOS computers. A previous ML model created by the authors designed to count *Plasmodium falciparum*-infected human RBCs did not perform well counting *Plasmodium*-infected mouse RBCs. We leveraged that model by loading the pretrained weights and training the algorithm with newly collected data to target *Plasmodium yoelii-* and *Plasmodium berghei-*infected mouse RBCs. This new model reliably measured both *P. yoelii* and *P. berghei* parasitemia (*R*^2^ = 0.9916). Additional rounds of training data to incorporate variances due to length of Giemsa staining and type of microscopes, etc., have produced a generalizable model, meeting WHO competency level 1 for the subcategory of parasite counting using independent microscopes. Reliable, automated analyses of blood-stage parasitemia will facilitate rapid and consistent evaluation of novel vaccines and antimalarials across laboratories in an easily accessible in vivo malaria model.

## INTRODUCTION

Eradication of malaria remains a global health priority, with 247 million cases and 619,000 deaths in 2021.^1^ Despite the scope of this problem, there is not yet a highly effective vaccine for malaria.^2^^–^^4^ The last few years have seen a drastic spike in malaria cases,^1^ and resistance is quickly spreading against the most effective therapeutics.^5^^–^^7^ Rapid population growth in countries where malaria is endemic, climate-driven epidemiological shifts in Africa,^8^ and the sudden reappearance of malaria in the American South^9^ provide further urgency for finding novel malaria treatments.

Research in blood-stage malaria has an essential and growing role in developing malaria vaccines and therapeutics for disease eradication. There is an urgent need for a blood-stage vaccine to reduce clinical malaria symptoms. Furthermore, the spread of artemisinin resistance has accelerated the need for novel antimalarials.^5^^–^^7^ Additionally, monoclonal antibodies capable of neutralizing blood-stage malaria are receiving heightened interest.^10^^–^^13^ In vivo rodent malaria models are likely to have a critical role in the development and evaluation of such new therapeutic tools.

The gold standard for analyzing rodent models of malaria involves manual counting of parasites in Giemsa-stained thin blood smears. However, this process is both time intensive and prone to human error. Alternative methods, including flow cytometry detection of nuclear stained parasite-infected red blood cells (iRBCs) or use of parasite strains expressing luciferase, have been reported.^14^ However, such approaches require specialized equipment and significant optimization and may be limited to specific parasite strains (e.g., parasites expressing fluorescent protein or luciferase). Additionally, they are incapable of collecting the wealth of information found on a blood smear such as infection within reticulocytes versus normocytes and the effect on the stage of the parasite (e.g., gametocytes). Additionally, counting parasitemia is well suited for automation, as it requires only common laboratory equipment and can significantly reduce the time spent determining parasitemia.

Several groups have previously sought to automate the analysis of human blood smears for malaria.^15^^–^^22^ Yu et al. developed an on-device smartphone-based system.^15^^,^^23^ It featured a mobile application, Malaria Screener, that was able to process the images captured through the eyepiece of a microscope and gave patient-level diagnosis. Another group developed a fully automated end-to-end system, EasyScan Go.^24^^,^^25^ It is a digital malaria microscopy device that is able to take a blood film slide, analyze it, and output its diagnosis all at once. Liu et al. developed an artificial intelligence-based system, AIDMAN.^16^ The findings of their research showed that deep learning models combined with image processing methods can detect and classify parasites with high accuracy.

However, human malaria species, such as *Plasmodium falciparum,* do not infect rodent RBCs, and such machine learning (ML) models may not be applicable to detection of rodent malaria. Three distinct *Plasmodium* species, *P. yoelii*, *P. berghei*, and *P. chabaudi,* which are evolutionarily related to *P. falciparum*, infect rodents. Machine learning researchers have historically focused on human malaria instead of rodents. Only two publications were found that focused on rodent malaria models. Ma et al. proposed a non-deep learning method.^26^ It did not recognize the morphology of parasites; instead, it used only the color of the Giemsa staining.^26^ Poostchi et al. also proposed a non-deep learning method, trained on *P. falciparum* iRBCs, and applied it to both human and mouse malaria with only modest success against the latter.^27^ The large functional conservation of proteins between *P. falciparum* and the rodent malaria species and availability of efficient tools for genetic manipulation make them an attractive model for in vivo evaluation. However, morphological differences in both host and parasites present difficulties when deep learning models trained to identify *P. falciparum*-infected human RBCs are directly utilized for rodent malaria models.

This work used a deep learning model pretrained for *P. falciparum-*infected human RBCs^15^ and fine-tuned it on 826 images of *P. yoelii*-infected RBCs from mice to develop an ML counting tool for *P. yoelii* and *P. berghei,* achieving an average relative error of 10.74% and 8.31%, respectively. To account for expected domain shifts between laboratories, we retrained the software to account for possible variations due to staining time, microscope, objective, and image acquisition platforms, etc., to produce a more generalizable model meeting WHO competency level 1 for parasite counting.^1^ Finally, we developed a Windows- and MacOS-compatible desktop application, Malaria Screener R, which embedded the developed deep learning model. This application lets users process blood smear images in batches and presents detection results with color labels drawn on the images. It also provides a convenient manual correction feature for mislabeled cells and saves the results to an Excel™ spreadsheet with graphical representations. Overall, we show that this platform can greatly enhance the capacity to efficiently and accurately evaluate preclinical malaria vaccine candidates and therapeutics across different laboratories.

## MATERIALS AND METHODS

### Overview.

In this project, we developed a system to automate the counting of *Plasmodium*-infected RBCs in rodents by using a deep learning-based algorithm. The YOLOv5 object detection model was used for this task. A software application, Malaria Screener R, was developed and used to process blood smear images, review the processed images with overlaid detection results, and export the parasitemia measurements to an Excel file.

The software is designed for use with any camera-equipped microscope. Typically, images captured with these microscopes are stored on a connected computer. For convenience, it is recommended to install Malaria Screener R on the same computer to eliminate the need for image transfer. Once images are captured, users should open the software and select the folder where the images are stored. The software will then process the images accordingly.

On average, our software was able to process one image in 2 to 3 s on one testing machine (2015 MacBook Pro; processor: 2.7-GHz dual-core Intel Core i5; memory: 8-GB, 1,867-MHz DDR3). Since our software typically needs about three images to achieve a good parasitemia estimation, this means it would take less than 10 s to finish one slide. Such performance can be achieved or surpassed by MacOS and Windows machines with similar or better computing power.

### Software development.

Malaria Screener R was built on top of an existing software called LabelImg.^28^ LabelImg is a software commonly used by the deep learning community for data labeling, a process where a human prepares the raw data for training by marking images with the ground truth information. While the valuable features of LabelImg, such as image browsing and image annotation, were retained, additional functionalities were incorporated. The new data selection function can handle folders with multilayer structures, which allows a large dataset with multilayer subfolders to be processed in a one-click fashion. The software will also use folder structure information to organize the results. An image analysis function was added using the aforementioned deep learning model. Furthermore, a user correction function allows quick rectification of model errors, such as mislabeled RBCs, during image browsing. Finally, a results display function was added to showcase processed image outcomes, with the option to export results to an Excel file where graphical representations are available. For example, if the dataset contains the data for multiple mice, one plot will have a bar graph to show the estimated percent parasitemia for each mouse. A user manual for software usage is provided in the supplemental data (Supplemental Figure 1). This software was written in Python. The deep learning model was trained using the Python-based PyTorch framework.

### Model training with YOLOv5.

YOLOv5 is a deep learning-based framework that performs object detection by processing the entire image in a single forward pass through the neural network, making it suitable for real-time applications such as the software application described in this work. YOLOv5 contains five different versions: YOLOv5n, YOLOv5s, YOLOv5m, YOLOv5l, and YOLOv5x. The main difference among these models is the amount of feature extraction modules. We chose the YOLOv5l version, which gave us a good balance between inference time and detection accuracy.

We used the hyperparameter evolution method provided in the YOLOv5 codebase to help choose the best hyperparameters for training. The training configurations and hyperparameters used for training the rodent models are provided in Supplemental Table 1. Model training was performed with a combination of graphics processing units, including the NVIDIA Tesla P100 and V100 models.

### Training and testing Model_Rodent_RBCs using images of *P. yoelii*-infected RBCs.

Deep learning is a data-intensive approach, requiring a large amount of human annotated data to achieve good results. Therefore, pretrained models are often used to save time and resources. A pretrained model is one that has been trained on large datasets. It can be used as a base model to be fine-tuned for a different but similar task. This will give the benefits of not needing as much data as training a model from scratch.

In this study, a model for detecting *P. falciparum* malaria in humans, which we termed Model_Human_RBCs, was used as the pretrained model. This model was developed using a dataset with 965 images from 200 patients collected in Bangladesh. Human and rodent parasite species have similar features. Therefore, the pretrained model for human malaria may be converted for rodent malaria after fine-tuning with additional training images from rodents. An additional 826 images were collected from 10 mice, all infected with *P. yoelii*, and used as training data for the new model, Model_Rodent_RBCs.

Training data were collected in the following manner. Blood smears were made from mouse tail bleeds, which were fixed and then stained with Giemsa (Sigma-Aldrich, St. Louis, MO; catalog no. 32884-1 L) for 10 min. Images were taken of blood smears on a Leica ICC50W microscope with a built-in camera using a 100× objective. Images were saved as tagged image file format (TIFF) files in red-green-blue (RGB) format with dimensions of 2,592 × 1,944 pixels. One parasitologist annotated all images using the Python application LabelImg.^28^ All cells were labeled as infected and uninfected, whereas nonspecific stains were labeled as debris.

For testing, images of *P. yoelii* and *P. berghei* were compared with manual counts of images. To test the labeling of *P. yoelii* with Model_Rodent_RBCs, 250 images from nine different mice and 26 unique blood smears were used. To test the labeling of *P. berghei* with Model_Rodent_RBCs, 43 images from four different mice and 11 unique blood smears were used. Enough images were taken for each smear to count at least 500 total RBCs for each unique smear (3 to 10 images per smear). No mice used in the test set were used in the training set. No user corrections were done after the automated analysis of the data.

### Training and testing Model_Rodent_RBCs_>10 min using darkly stained images.

Increased staining time causes darker nonspecific staining and a higher chance of false positives. To improve the accuracy for such images, 100 additional images were collected to retrain the model. All images came from blood smears of *P. berghei*-infected mice. Training data were collected and annotated in the manner described above with the following modification: blood smears were stained with Giemsa for either 10, 20, or 70 min. The longer-stained images were mixed into the training data together with the images from the 10 mice infected with *P. yoelii*. Training was repeated using the same pretrained *P. falciparum* model, Model_Human_RBCs. Training was done in the same manner as described above for Model_Rodent_RBCs. However, the original 826 images plus the new 100 images were used to train this model, which we termed Model_Rodent_RBCs_>10 min.

For testing, blood smears were taken from *P. berghei*-infected mice and allowed to stain in Giemsa stain (Sigma-Aldrich; catalog no. 32884-1 L) for 10, 30, or 50 min before viewing. A total of 20 smears were analyzed (*N* = 7, 7, and 6 for 10-, 30-, and 50-min groups, respectively). The same smears were used for analyses by both Model_Rodent_RBCs and Model_Rodent_RBCs_>10 min. Enough images were taken to gather >500 total RBCs for unique blood smears. No mice used in the test set were used in the training set. No user corrections were done after the automated analysis of the data. Manual counting of images was used as a reference for comparing the accuracy of the two automated models, Model_Rodent_RBCs and Model_Rodent_RBCs_>10 min.

### Training and testing Model_Rodent_RBCs_New_Micro using images from various microscopy platforms.

To make the model more generalizable in real-world settings, 99 additional images were collected from three different microscopy platforms to retrain the model, creating Model_Rodent_RBCs_New_Micro. Fifty-four images came from blood smears of *P. berghei*-infected mice, and 45 images came from blood smears of *P. yoelii*-infected mice. Training data were collected on a Nikon E800 microscope with a Spot RT Slider top-mounted camera using a 100× objective and on a Nikon E600 microscope with a DS-Ri1 top-mounted camera with both 100× and 40× objectives. Blood smears were stained with Giemsa for 10 min. All files were saved as TIFF files in RGB format. For the Nikon E600 with a 40× objective, images were saved with dimensions of 1,280 × 1,024 pixels. For the Nikon E600 with a 100× objective, images were saved as 1,920 × 1,440 pixels. Finally, for the Nikon E800, images were saved as 1,600 × 1,200 pixels. Again, these new images were mixed into the training data. Then, the model was retrained by repeating the training setup from the previous model, Model_Rodent_RBCs_>10 min. This final updated model created from this training set was termed Model_Rodent_RBCs_New_Micro.

Testing was performed in the following manner. For the Nikon E800 with a 100X objective, automated and manual counts were compared using images from four different mice and 10 unique blood smears. A total of 36 images were used for these 10 blood smears. For the Nikon E600 with a 40× objective, automated and manual counts were compared using images from six different mice and 10 unique blood smears. A total of 16 images were used for these 10 blood smears. Finally, for the Nikon E600 with a 100× objective, automated and manual counts were compared using images from six different mice and eight unique blood smears. A total of 77 images were used for these eight blood smears. Enough images were taken to gather more than 500 total RBCs per unique blood smear. No mice used in the test set were used in the training set. No user corrections were done after the automated analysis of the data.

### Parasitemia estimates by expert parasitologists.

Expert parasitologists consisted of PhD students and postdoctoral fellows who had completed more than 3 years of malaria research. Parasitemia estimates by the four expert parasitologists were performed in the following manner. First, the total number of RBCs in one field was counted. Then, the number of infected RBCs in the same field was counted. Next, the stage was moved vertically to the next field, which was assumed to have a similar distribution of RBCs, and the numbers of infected and total RBCs were counted. This process was then repeated for 10 fields. The number of total RBCs was estimated by averaging the counts from the first and last fields examined and then multiplying this average by 10, the number of fields counted.

### Manual counting of individual RBCs in images.

The same images uploaded into the automated program were viewed by an expert parasitologist. With the assistance of the application ScreenGrid^TM^, each infected and uninfected RBC within the image was counted. For cells on the border of images, only those cells where the majority of the cells were present in the image frame were included in counts. Likewise, cells largely obscured by debris were not included in counts. Infected and uninfected RBCs were counted two times, and the average of the two values was used. If the two counts varied by more than 10%, a third count was done.

### Infection of mice.

Female C57Bl/6 mice (Jackson Laboratory, Bar Harbor, ME) and female Swiss Webster mice (Charles River Laboratories, Newark, DE) of various ages (4 to 14 weeks) were used in this study. Mice were infected via the tail vein with 10^4^ infected RBCs, containing either the *P. yoelii* or *P. berghei* parasite species. Mice were euthanized if parasitemia reached 75% or higher.

### Parasite strains.

Both nonlethal *P. yoelii* XNL and lethal *P. yoelii* 17XL strains were used for *P*. *yoelii* infections. *P. berghei* strain ANKA parasites were used for *P. berghei* infection.

### Production and staining of blood smears.

Mouse tail bleeds were performed daily starting 2 to 3 days postinfection. A small drop of blood was placed on a glass slide, and a smear was made. Smears were placed for a few seconds in 100% methanol for fixation and then in Giemsa stain (Sigma-Aldrich; catalog no. 32884-1 L) for 10 min, unless otherwise noted within the study. Slides were allowed to air dry, or in some cases, a hair dryer was used to speed up the drying process. Slides were then viewed underneath a light microscope.

## RESULTS

### Blood smear estimates of *P. yoelii* and *P. berghei* parasites by parasitologists are highly variable.

**Figure 1.**
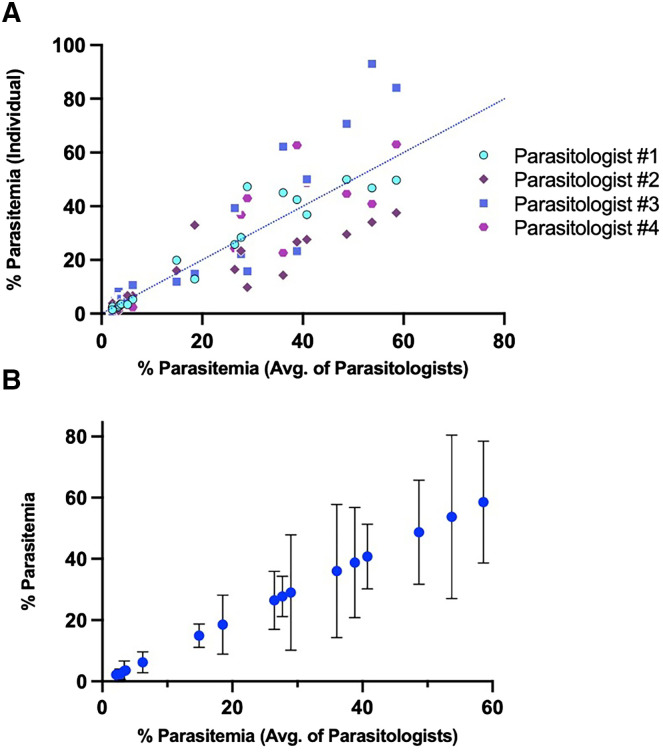
Parasitemia estimates from blood smears are highly variable. (**A**) Estimates of percent parasitemia from individual parasitologists are shown as single points (*y* axis) plotted against the average parasitemia measurement of all four parasitologists (*x* axis). A blue dotted line represents the position of parasitemia measurements if equivalent to manual counts. (**B**) Blue circles represent the percent parasitemia of a single mouse at a single time point (*y* axis). Error bars represent SD. Avg. = average.

Four parasitologists were tasked with performing estimates of parasitemia on thin blood smears using the same 19 Giemsa-stained smears of *P. yoelii-* and *P. berghei*-infected RBCs, 10 with *P. berghei* and 9 with *P. yoelii.* Each parasitologist used a standard system for measuring parasitemia, counting all infected RBCs in each field and estimating the total number of RBCs in each field (detailed further in Materials and Methods). Parasitemia varied significantly between parasitologists (Figure 1A and B), with an average relative SD of 43.31% among the 19 blood smears tested (Supplemental Figure 2). Significant deviation was found at all measurable parasitemia levels, and SD showed no correlation with parasitemia levels (*R*^2^ = 0.0099) (Supplemental Figure 2).

### Previous *P. falciparum* trained model fails to accurately measure parasitemia in *P. yoelii-*infected rodent RBCs.

**Figure 2.**
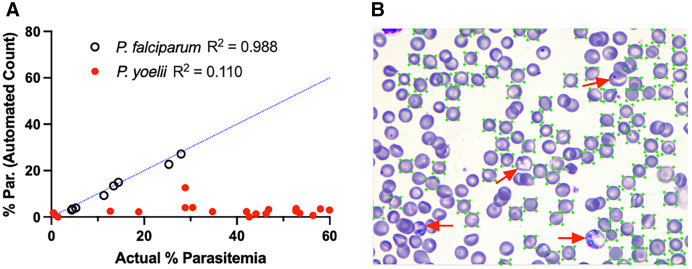
Previous model trained on *Plasmodium falciparum*-infected human red blood cells (RBCs) fails to detect *Plasmodium yoelii*-infected mouse RBCs. (**A**) Each circle represents a parasitemia measurement determined using the previous *P. falciparum*-trained malaria screener model trained on *P. falciparum*-infected RBCs (black open circles) or *P. yoelii*-infected RBCs (red closed circles). The *x* axis represents percent parasitemia (% Par.) measured by manual counting of RBCs in images by an expert parasitologist. A blue dotted line represents the position of parasitemia measurements if equivalent to manual counts. (**B**) Sample image of automated detection of RBCs using this model. Green boxes circumscribe all RBCs (both infected and uninfected) recognized by the model. Red arrows indicate infected RBCs not recognized by the software.

Previously, our group developed and optimized a ML model (Model_Human_RBCs) to identify *P. falciparum*-infected human RBCs in Giemsa-stained thin smears from patients in a clinical setting.^15^ In the current study, this model was applied to measure both *P. falciparum*-infected human RBCs and *P. yoelii*-infected mouse RBCs. Although the program accurately measured parasitemia in *P. falciparum*-infected human RBCs, it failed to do so in *P. yoelii*-infected mouse RBCs (Figure 2A). When Model_Human_RBCs was tested on *P. falciparum*-infected RBCs, automated counts correlated highly with manual counts (*R*^2^ = 0.988) (14.25% relative error rate). In contrast, when Model_Human_RBCs was tested on *P. yoelii*-infected RBCs, automated counts correlated poorly with manual counts (*R*^2^ = 0.110) (100.83% relative error rate). On the *P. yoelii*-infected RBC test set, the model underperformed in two areas, failing to identify mouse RBCs (both infected and uninfected) and to differentiate infected from uninfected mouse RBCs (Figure 2B).

### Model trained on images of *P. yoelii*-infected RBCs accurately estimates rodent parasites at a wide range of parasitemia and RBC densities.

**Figure 3.**
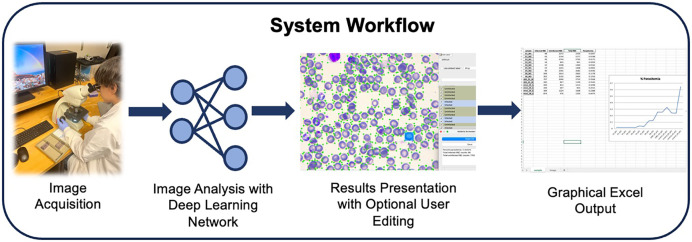
Schematic demonstrating workflow of automated malaria detection system. Images of Giemsa-stained rodent thin blood smears are captured using a light microscope with a built-in camera. Images are uploaded to software, and each cell is detected and then labeled as infected or uninfected using a pretrained convolutional neural network. Each annotated image is then displayed in a graphical interface allowing users to verify and, if necessary, to correct labels. After each image is saved, an Excel sheet is created with a graphical output of percent parasitemia measurements. A graphical summary of results is organized using the directory structure of uploaded images and user input of groupings upon saving.

**Figure 4.**
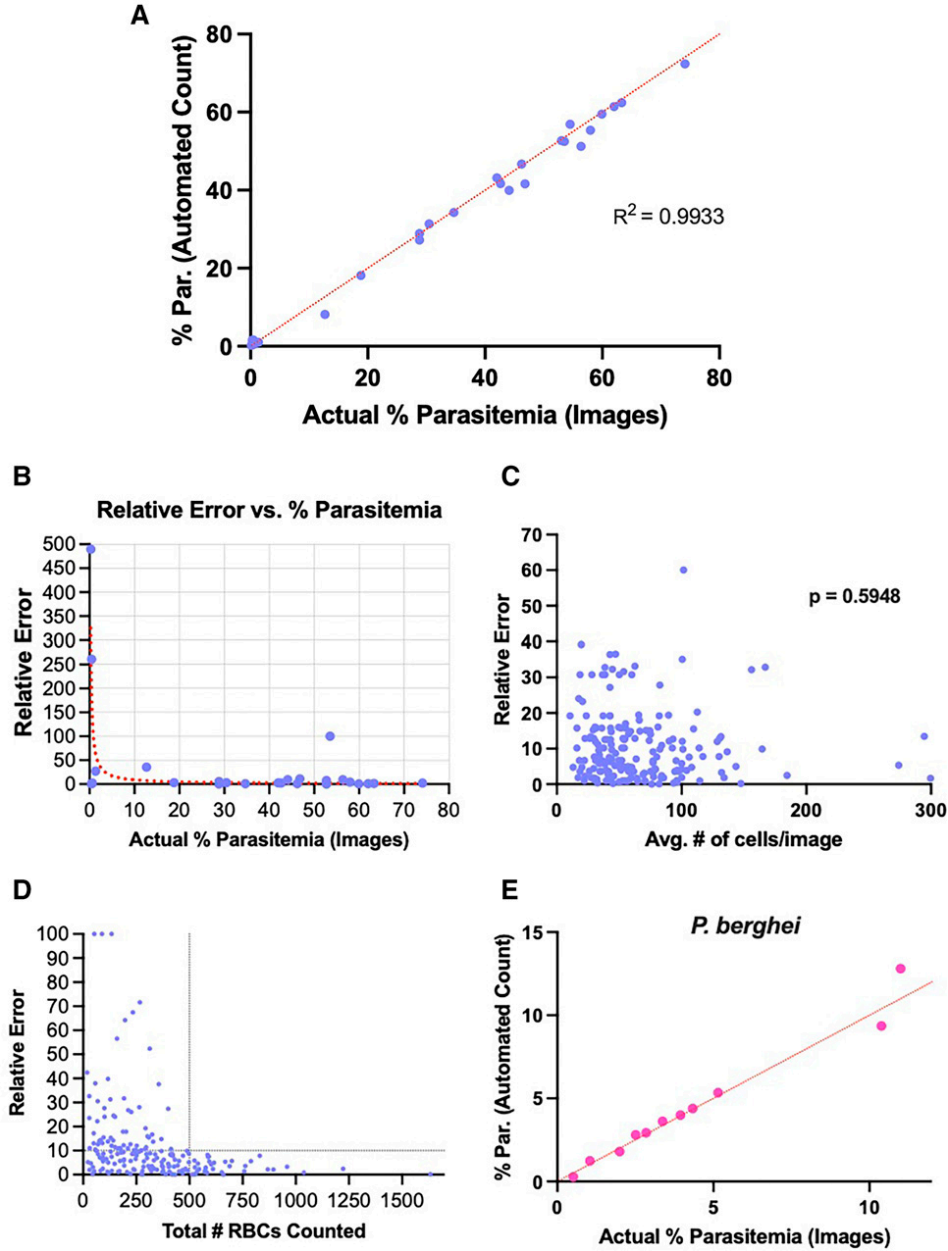
Automated detection method calculates percent parasitemia of *Plasmodium yoelii-* and *Plasmodium berghei-*infected cells with high accuracy. (**A**) Relationship between manual counting of parasitemia (*x* axis) and percent parasitemia measured using the automated detection method. Each blue dot represents the percent parasitemia of a *P. yoelii*-infected mouse at a single time point. A red dotted line indicates the position of each point if the manual and automated counts are equal. (**B**) Relative error of automated parasitemia measurements of *P. yoelii*-infected mice. Blue dots represent relative errors between automated and manual measurements for one mouse at one time point utilizing Model_Rodent_RBCs. The relative error is calculated as the absolute value of [(% parasitemia by automated count – % parasitemia by manual count)/(% parasitemia by manual count)] × 100. A red dotted line represents nonlinear fit of relative error versus percent parasitemia. (**C**) Relationship between relative error (*y* axis) and red blood cell (RBC) density (*x* axis). Each blue dot represents the total number of RBCs in a single image plotted against the relative error between manual and automated counts for that image. (**D**) Relationship between relative error (*y* axis) and total number of RBCs (infected and uninfected) counted by automated software. Blue dots represent the total number of cells counted by the software and the relative error compared with the count by manual measurements. Data points are based on parasitemia measurements of one or more images of blood smears from a single mouse at a single time point. Additional images from the same blood smears were added in series. Intersection of black dotted lines indicates stabilization of relative error values around 500 total RBCs counted. (**E**) Pink dots show the relationship between manual and automated parasitemia measurements of *Plasmodium berghei-*infected RBCs. A red dotted line shows the position of points if the measurements are equal.

Because of the limitations of the previous model trained on *P. falciparum*-infected human RBCs in identifying *P. yoelii*-infected mouse RBCs, we sought to develop a new model capable of labeling such cells, which we termed Model_Rodent_RBCs (Figure 3). We fine-tuned the model using 826 images taken from 10 different *P. yoelii*-infected mice at different parasitemia levels (Figure 3). This led to a substantial improvement in identification of uninfected and infected RBCs, with the automated counts closely matching the manual counts determined from the same set of images (*R*^2^ = 0.9933) (Figure 4A and Supplemental Figure 3). For parasitemia levels greater than 1%, the relative error was low between automated and manual counts (mean relative error = 10.74%) (Figure 4B and Supplemental Figure 3). To evaluate accuracy, we created two different percent parasitemia reference standards (Supplemental Figure 3A). The first was based on manual estimates of parasitemia in blood smears. In this method, typically used for parasitemia measurements, the total number of cells in each microscopy field is estimated from a subset of fields [% parasitemia = (iRBCs in 10 fields)/(RBCs in 2 fields picked at random × 5)]. In the second method, we counted every cell in the images directly used for the test data, a more stringent and time-consuming approach (% parasitemia = total iRBCs/total RBCs). Whereas the former standard helps validate the accuracy in a more real-world setting, the latter provides a more precise manner for determining the accuracy of the model in classifying cells in the images analyzed. Notably, we saw improvements in the accuracy of the automated system versus a manual count when using comparisons against both standards (Supplemental Figure 4A to C). In a comparison against manual estimates of parasitemia in blood smears, the automated system demonstrated better accuracy than manual estimates by three of four expert parasitologists and accuracy equivalent to that of the fourth parasitologist. In a comparison against an individual count of RBCs in images, the automated system demonstrated better accuracy than the counts by all four parasitologists.

At parasitemia levels >1%, the program produced highly accurate counts (median relative error = 5.88%). However, at parasitemia levels <1%, the error rate was higher (74.78% median relative error) (Figure 4B). At very low parasitemia levels, false positives can lead to a high error rate. Indeed, the errors observed were typically characterized by the presence of one or more false-positive infected RBCs in the automated detections. Our ML software, Malaria Screener R, provides the user the ability to easily correct the label on only a few RBCs to correct such false positives and account for these errors.

Overlapping RBCs may often be difficult for automated software to distinguish. To test the model’s ability to handle such situations, we analyzed the *P. yoelii*-trained model’s (Model_Rodent_RBCs) performance at a range of cell densities, up to 300 RBCs per field, using a Leica DMi8 microscope with an integrated camera. The Model_Rodent_RBCs model performed similarly across all tested RBC densities (Figure 4C). There was not a significant relationship between the number of RBCs per image and the relative error (Pearson correlation coefficient = 0.03853, *P* = 0.5948). Importantly, error rates for the program tended to decrease as the number of RBCs analyzed increased. To better inform the user’s decision of how many images to upload to the software, we analyzed the relationship between relative error of the program and the total number of RBCs analyzed. Relative error between automated and manual counts appeared to stabilize below 10%, around 450 to 500 total RBCs counted (Figure 4D). This value corresponds to about three images taken containing 150 RBCs per image, a density which we previously demonstrated the program can reliably measure (Figure 4C).

*Plasmodium yoelii* and *P. berghei* are two commonly used rodent malaria parasites in preclinical studies. As the dataset for fine-tuning the model used only *P. yoelii*-infected RBCs, we tested the program’s ability to measure parasitemia from 11 different blood smears of *P. berghei*-infected mice at parasitemia values ranging from 0.5 to 11%. The software accurately measured parasitemia across a range of values with a median relative error of 8.33% for parasitemia values >1%, indicating that the program performs comparably on both rodent malaria parasites (Figure 4E).

### Giemsa stain time of blood smears has a significant effect on model performance.

When comparing manual and automated counts, we noticed that the accuracy of the program was affected when different individuals performed the Giemsa staining of blood smears. Examining this issue further, we found that the difference was related to a change in the length of time slides were stained with Giemsa. For slides stained for 10 to 15 min, the normal recommendation, the program annotated with high accuracy. However, when the stain time was extended to 30 to 50 min, the accuracy of the program decreased drastically, as increased nonspecific staining led to a higher number of falsely labeled infected RBCs (Supplemental Figure 5A). To account for such potential differences, we trained a separate model with an additional 100 new images from blood smears stained for atypically long times (Model_Rodent_RBCs_>10 min). After retraining, there was a decrease in error rates for blood smears stained for 30 and 50 min (Supplemental Figure 5B). However, error rates for these extended stain times remained much higher than error rates for images from smears stained for 10 min. Interestingly, accommodating for longer staining protocols also improved the performance of the program at parasitemia values below 1% to a median relative error of 17% compared with 35% before optimization (Supplemental Figure 5C). Therefore, it is recommended not to stain longer than 10 min.

### Model is generalizable to different microscopes and image-acquisition platforms.

**Figure 5.**
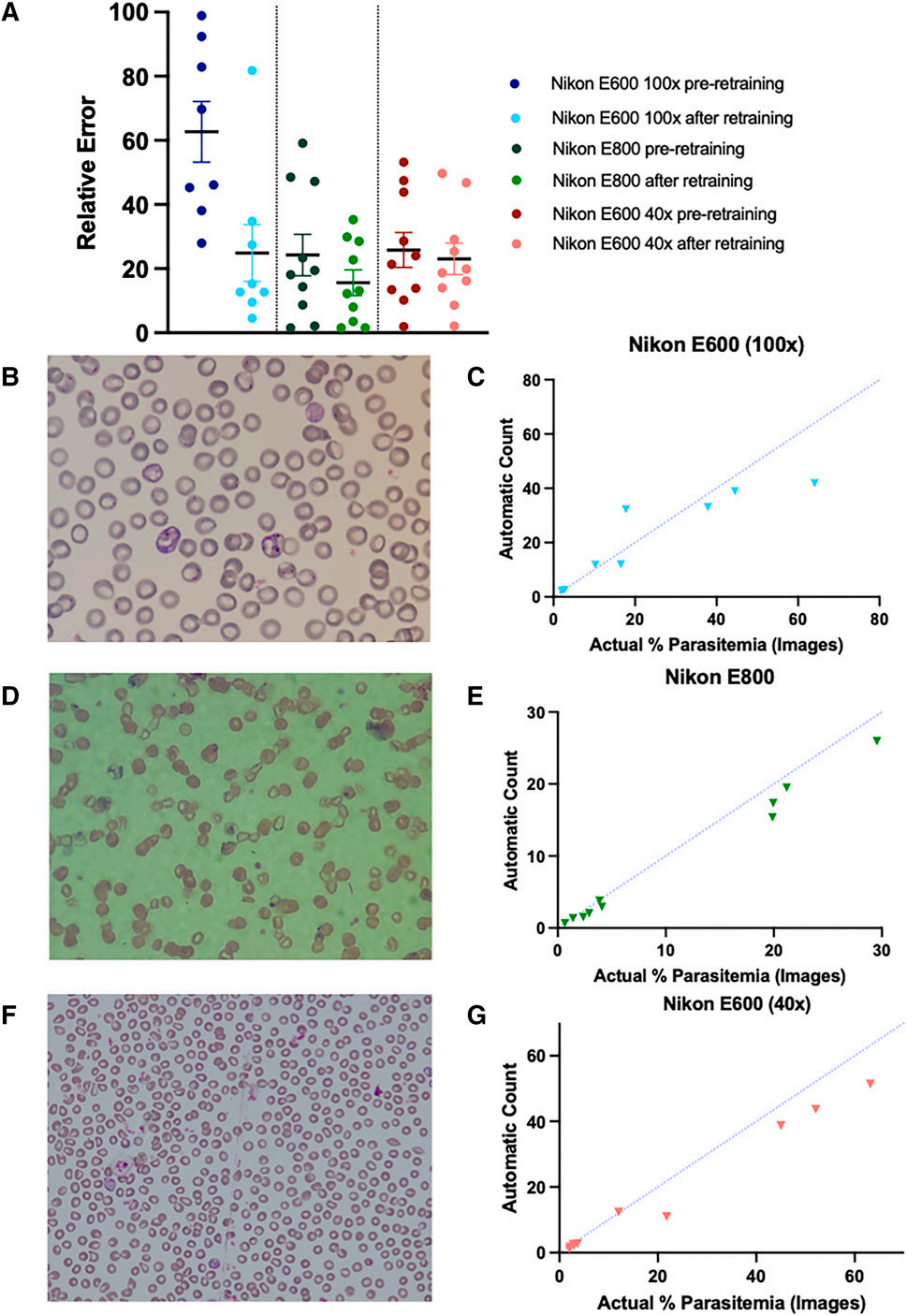
The model is generalizable to images captured on different microscopes. (**A**) Relative error (*y* axis) is shown for the difference between the original model’s annotations and manual counts using images from blood smears on a Nikon E600 microscope with a 100× objective (dark blue, left), on a Nikon E800 microscope with a 100× objective (dark green, center), and on a Nikon E600 microscope with a 40× objective (dark red, right). Relative error was calculated for each mouse at one time point as the absolute value of [(% parasitemia by automated count – % parasitemia by manual count)/(% parasitemia by manual count)] × 100. The model was then retrained with an additional 99 images total from these microscopes and updated to a single new model, Model_Rodent_RBCs_New_Micro. Relative error was recalculated for each data point and is displayed in light blue, light green, and light red for the Nikon E600 (100× objective), Nikon E800 (100× objective), and Nikon E800 (40× objective) microscopes, respectively. Data are mean ±SEM. (**B, D, and F**) Sample images of blood smears taken on a Nikon E600 with a 100× objective (**B**), on a Nikon E800 with a 100× objective (**D**), and on a Nikon E600 with a 40× objective (**F**). (**C, E, and G**) Relationship between manual counting of parasitemia (*x* axis) and percent parasitemia measured by the automated detection method, using images taken on a Nikon E600 with a 100× objective (**C**), on a Nikon E800 with a 100× objective (**E**), and on a Nikon E600 with a 40× objective (**G**). Each dot represents the percent parasitemia of a *Plasmodium yoelii-* or *Plasmodium berghei*-infected mouse at a single time point. A blue dotted line indicates the position of each point if manual and automated counts are equivalent.

Differences in cameras, microscopes, and objectives used may alter image quality and influence count accuracy. Indeed, images acquired using three different microscopes (Nikon E800 with a 100× objective, Nikon E600 with a 40× objective, and Nikon E600 with a 100× objective) varied significantly in quality and size of RBCs (Figure 5B, D, and F), and all three microscopes showed higher relative error values than the Leica microscope used for capturing images used for ML training in Model_Rodent_RBCs. This lower accuracy appears to be due to undercounting in 24 of 27 test cases. To address this domain shift, the model was fine-tuned using 99 additional annotated images collected from different microscopes, 54 images of *P. berghei* parasites and 45 images of *P. yoelii* parasites (Model_Rodent_RBCs_New_Micro). After fine-tuning, automated counts for all three microscopes fit more closely to expected values, determined by manual counting of individual cells in images (Figure 5A). Average relative error rates were 15.64%, 23.07%, and 24.84% for the Nikon E800 with a 100× objective, Nikon E600 with a 40× objective, and Nikon E600 with a 100× objective, respectively (Figure 5A, C, E, and G). The <25% error rate obtained for all three additional microscopes after training optimization reached level 1 competency when measured using WHO guidelines for assessment of microscopists in human malaria diagnosis.^29^

### Automated model predicts parasitemia with greater precision than manual counting.

**Figure 6.**
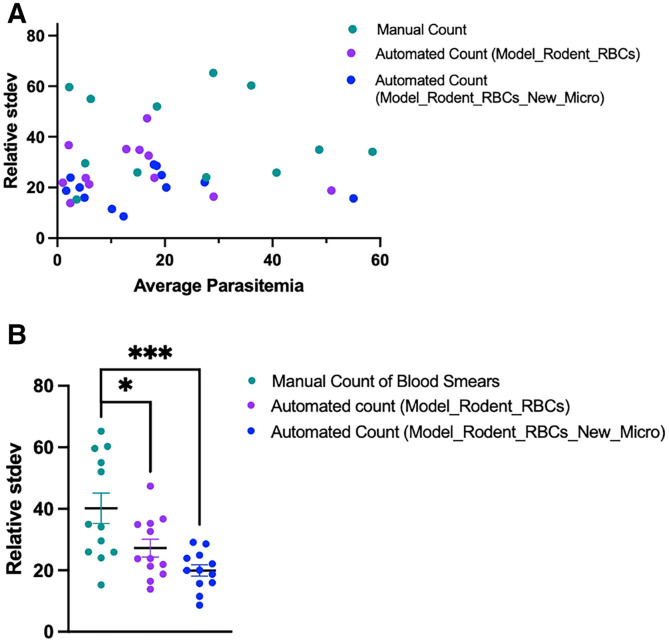
Automated model predicts parasitemia with greater precision than manual counting. (**A**) Relative SD (stdev) of parasitemia measurements (*y* axis) is shown for four different expert parasitologists manually counting blood smears (teal) and four parasitologists using the automated software trained on parasites infecting rodent RBCs (Model_Rodent_RBCs = purple, Model_Rodent_RBCs_New_Micro = blue) across a wide range of parasitemia measurements. *x* axis values denote the average of measurements by four users manually counting blood smears or using the automated software. Relative SD was calculated as (100 × SD)/(mean % parasitemia). (**B**) Relative SD for all parasitemia measurements is shown when four users manually count blood smears (teal) versus when four users use the automated software (Model_Rodent_RBCs = purple, Model_Rodent_RBCs_New_Micro = blue). *P* values were calculated using unpaired, two-tailed *t* tests.

Comparison of in vivo models of malaria both within and between laboratories requires a consistent means of measuring parasitemia. The large SD of parasitemia measurements between parasitologists counting the same blood smears (Figure 1A and B) hinders such comparisons. To evaluate precision of the automated platform versus manual counting of blood smears, four parasitologists were tasked with measuring the same 11 blood smears using standard manual counting and four users were tasked with measuring the same 11 blood smears using the automated method developed in this study (Figure 3). The automated program with both deep learning networks, Model_Rodent_RBCs and Model_Rodent_RBCs_New_Micro, demonstrated significantly greater precision, measured via relative SD, than parasitologists manually counting blood smears (Figure 6A and B). Across all parasitemia levels, the automated program showed greater precision than manual counting (Figure 6A and B). Interestingly, the automated method using the updated model, Model_Rodent_RBCs_New_Micro, showed even greater improvement over the original model, Model_Rodent_RBCs.

## DISCUSSION

Rodent models of malaria can play a critical role in testing preclinical therapeutics and vaccines for prioritizing subsequent evaluation in nonhuman primates or humans. However, the gold standard for analyzing blood-stage malaria in these models relies largely on counting of blood smears, a time-consuming and repetitive process. This method can also be error prone because of the use of estimates rather than actual counts of total RBCs and parasitologists’ biases in classifying infected versus uninfected RBCs. Parasitologists typically estimate the total number of RBCs they are counting, because individual counting of RBCs in all fields is too time-consuming. In this study, we find a remarkably high average relative SD in parasitemia measurements of 43.31% between expert parasitologists.

We used transfer learning to adapt an ML model, which was previously trained on *P. falciparum*-infected human RBCs, to identify *P. yoelii*-infected mouse RBCs. We developed a Windows- and MacOS-compatible desktop application incorporating this model and capable of providing accurate parasitemia measurements with limited user input. This software requires only a microscope with a mounted or built-in camera and a computer for analysis. Using this semiautomated process, the user will make blood smears, collect images on a microscope with the camera, and process the images with the software. The software then rapidly annotates each cell as infected or uninfected and provides both a table and graphical representation of the results in a spreadsheet.

In contrast to standard manual counting methods, which count infected RBCs but estimate the total number of RBCs, the automated program counts all uninfected and infected RBCs, theoretically allowing for more accurate measurements. Supporting this theory, the automated system demonstrates better accuracy than that of expert parasitologists’ manual counts of the same smears. This automated program (Model_Rodent_RBCs) demonstrated average relative errors of 10.74% and 8.31% compared with image-based manual counts for *P. yoelii* and *P. berghei*, respectively, across a wide range of RBC densities and parasitemia values (1% to 75%). Importantly, the model accurately identifies RBCs at very high densities, allowing for acquisition of only a few images to achieve robust sample sizes. In addition to improving accuracy, the model also provides significantly more consistent results. Counting all RBCs, as opposed to estimating total RBCs, and the elimination of parasitologists’ biases in parasite classification likely contribute to the improved consistency of the automated program over manual counting. This result, importantly, allows for effective comparison of different therapeutics across distinct rodent malaria studies.

The initial model, Model_Rodent_RBCs, trained using images from a single microscope, demonstrated a loss in accuracy when parasitemia was measured using images taken on different microscope platforms. Interestingly, after retraining with only the addition of a small number of images from different platforms, the model met accuracy levels comparable to the WHO level 1 standard for counting *P. falciparum* parasites.^29^ Remarkably, after switching from a 100× to a 40× objective and thereby drastically decreasing the apparent size of both RBCs and parasites, the model did not show a loss of accuracy. These results suggest broad applicability of this model to a variety of microscopes and laboratories.

During this study, we found that the Giemsa stain time of blood smears impacted the accuracy of the program, with longer than normal stain times leading to a significant loss of accuracy. Retraining with additional images from longer-stained smears was able to significantly improve the accuracy of measurements on such smears but still demonstrated a loss of accuracy. For this reason, we strongly recommend limiting Giemsa stain times to 10 to 15 min when using this program. Retraining, however, offered an unexpected benefit to the accuracy of the program. Model_Rodent_RBCs_>10 min demonstrated an improved ability to distinguish false positives. This improvement is most apparent for images with very low parasitemia values (<1%), where a single false positive may drastically affect the parasitemia and relative error. In Model_Rodent_RBCs_>10 min, there was a 4-fold decrease in the average relative error in parasitemia measurements for images below 1% parasitemia, in comparison with Model_Rodent_RBCs. Although the updated model still has only moderate accuracy below 1% parasitemia (median relative error, 16.97%), the software’s built-in function to optionally correct mislabeled RBCs offers a rapid means of correcting measurements at such low parasitemia values. All results in this study do not include optional corrections, and any such corrections will lead to greater improvements in accuracy.

The program developed in this study provides a consistent, accurate, and efficient method for the analysis of infected RBCs in rodent malaria models. Importantly, this ML-based automated tool, Malaria Screener R, can provide reliable parasitemia counts and enable fast evaluation of novel vaccines and antimalarials in an easily accessible in vivo malaria model. The training data used in this study has also been fully annotated with the parasite stage (gametocyte, ring, schizont, trophozoite), the number of parasites in each RBC, and the identification of both infected and uninfected immature RBCs (reticulocytes). Future updates to the software may incorporate staging parasites, separate counting of multiply infected RBCs, and identifying infected and uninfected reticulocytes.

## Supplemental Materials

10.4269/ajtmh.24-0135 Supplemental Materials
