# Supplementary material for: Application of Machine Learning in a Rodent Malaria Model for Rapid, Accurate, and Consistent Parasite Counts
Source: Am J Trop Med Hyg. 2024 Sep 10;111(5):967–76. doi: 10.4269/ajtmh.24-0135 (PMC11542515; doi:10.4269/ajtmh.24-0135)
Supplement: Supplemental Materials [file tpmd240135.SD1.pdf]

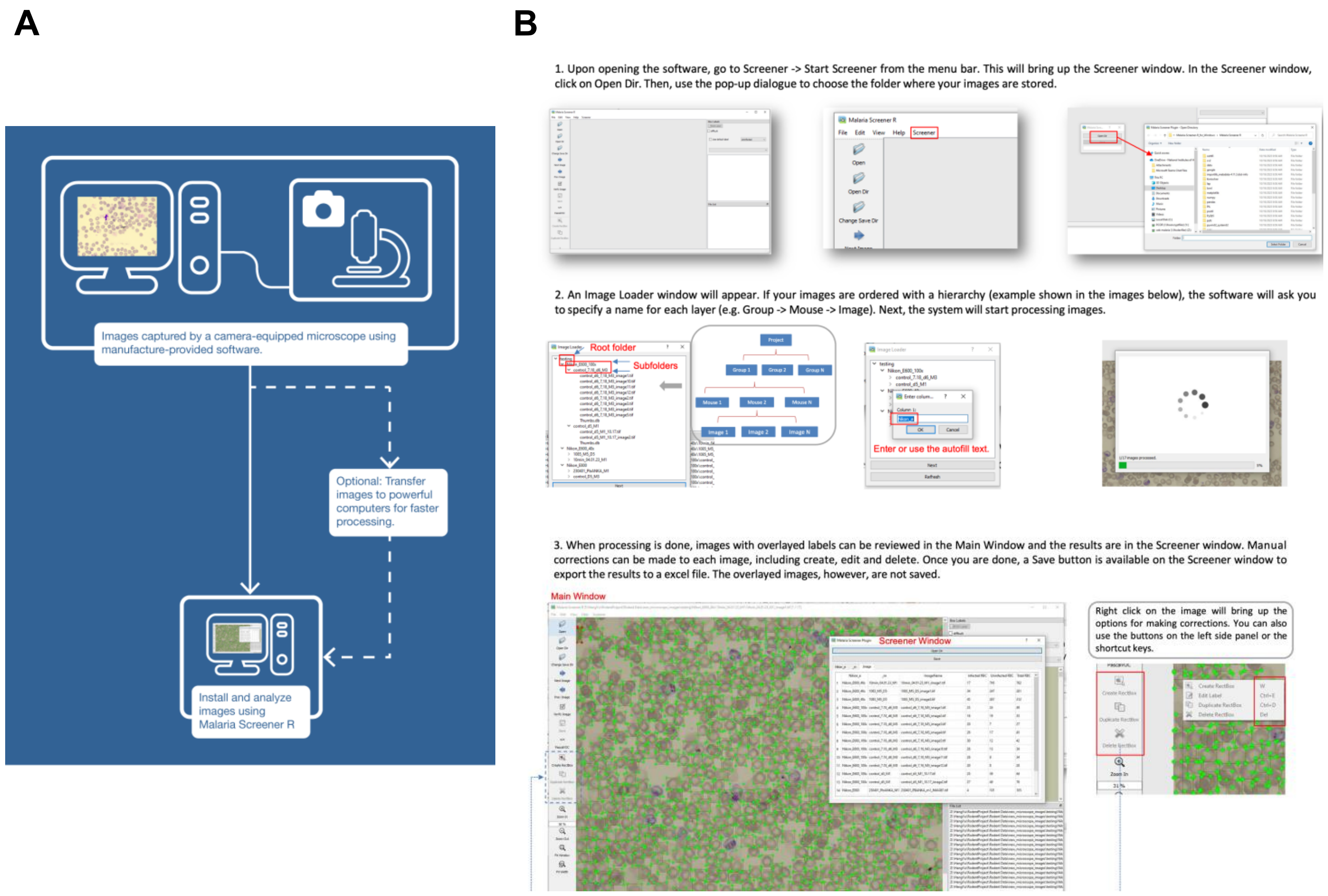

Supplementary Figure 1: User manual demonstrating proper usage of software for automated detection of *Plasmodium* infected rodent RBCs. A) A flow chart illustrates the steps involved in using Malaria Screener R. B) Steps 1-3 outline software usage for automated detection of RBCs from images captured under microscope.

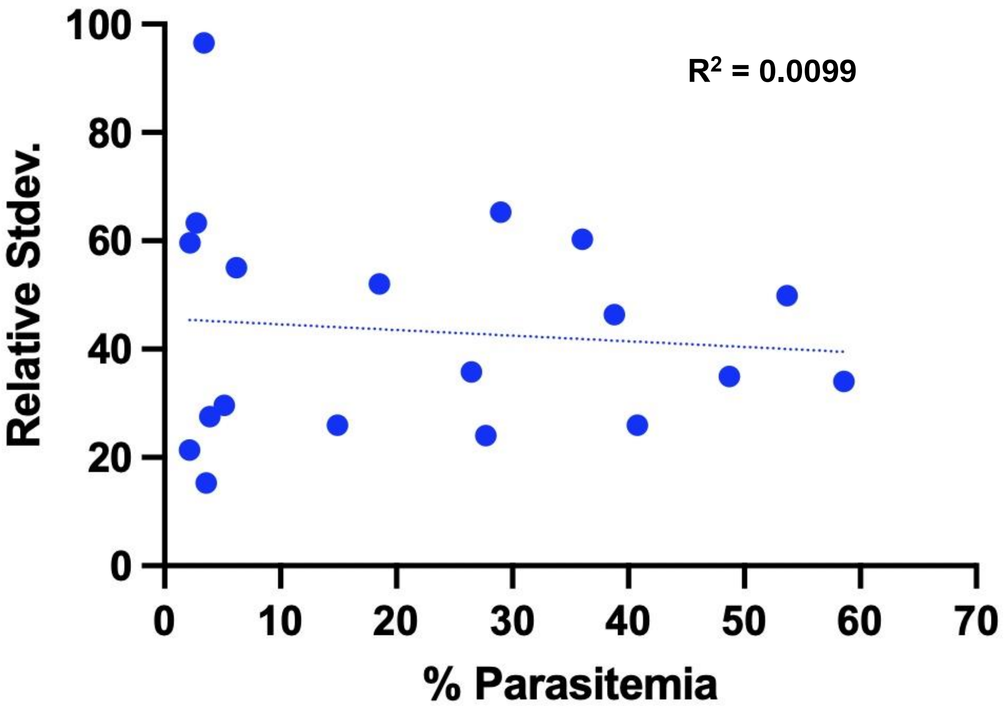

Supplementary Figure 2: Relative Standard Deviation vs. Percent Parasitemia. Blue circles represent the standard deviation (y-axis) between parasitemia estimates from 4 different parasitologist across a range of parasitemia values (x-axis). Blue dotted line is a simple linear regression of all data points. R^2 value is shown in the upper right.

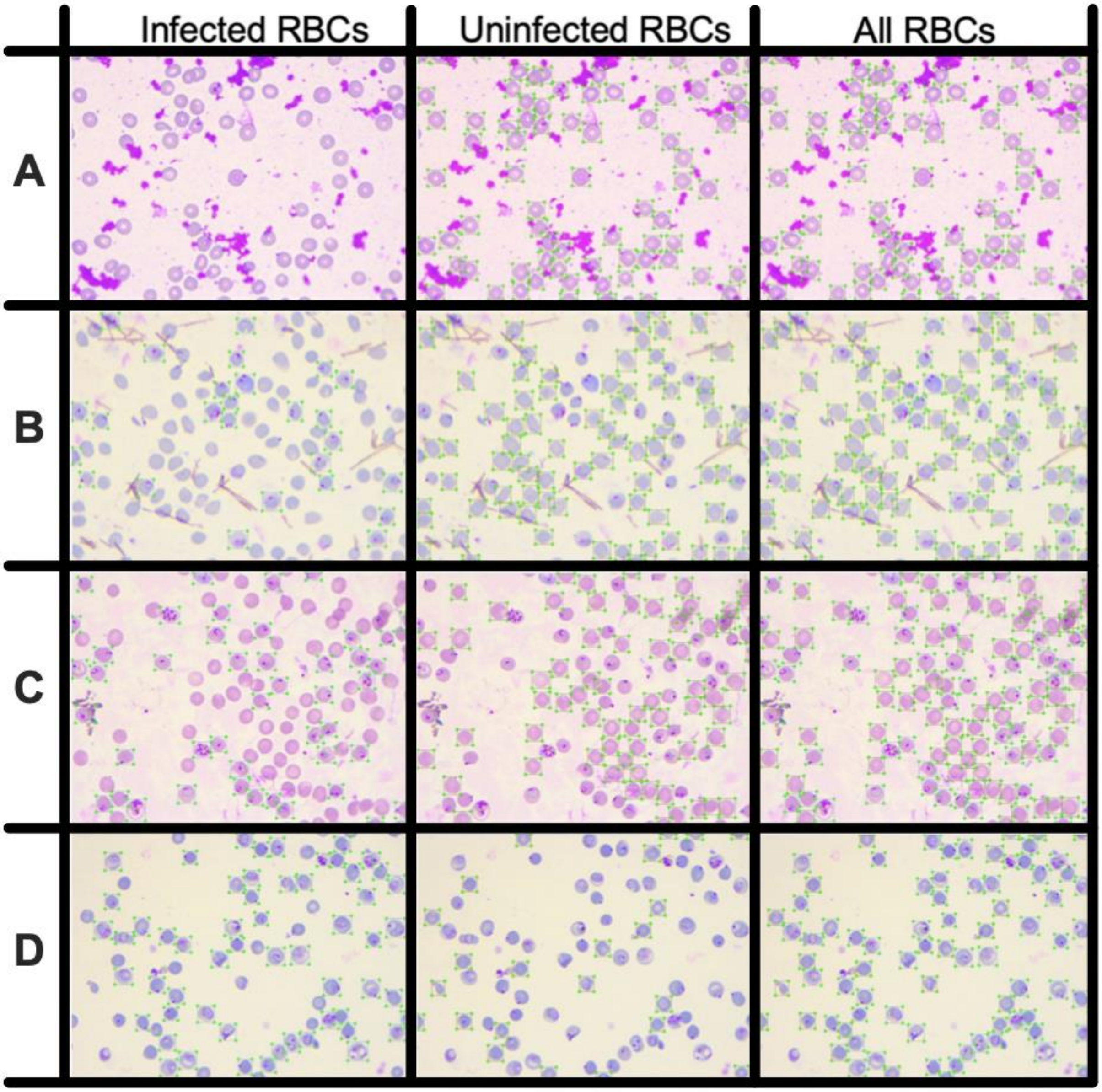

Supplementary Figure 3: Example images of model performance on *P. yoelii* infected rodent RBCs. Four example images of the model's (Model_Rodent_RBCs) automated detections are shown at 0 % (A), 25% (B), 39% (C), and 68% (D) parasitemia respectively. Left column highlights RBCs labeled by the model as infected. Center column highlights RBCs labeled by the model as uninfected. Right column

highlights all detected RBCs by the model. Green circumscribing boxes represent positive labels for each category.

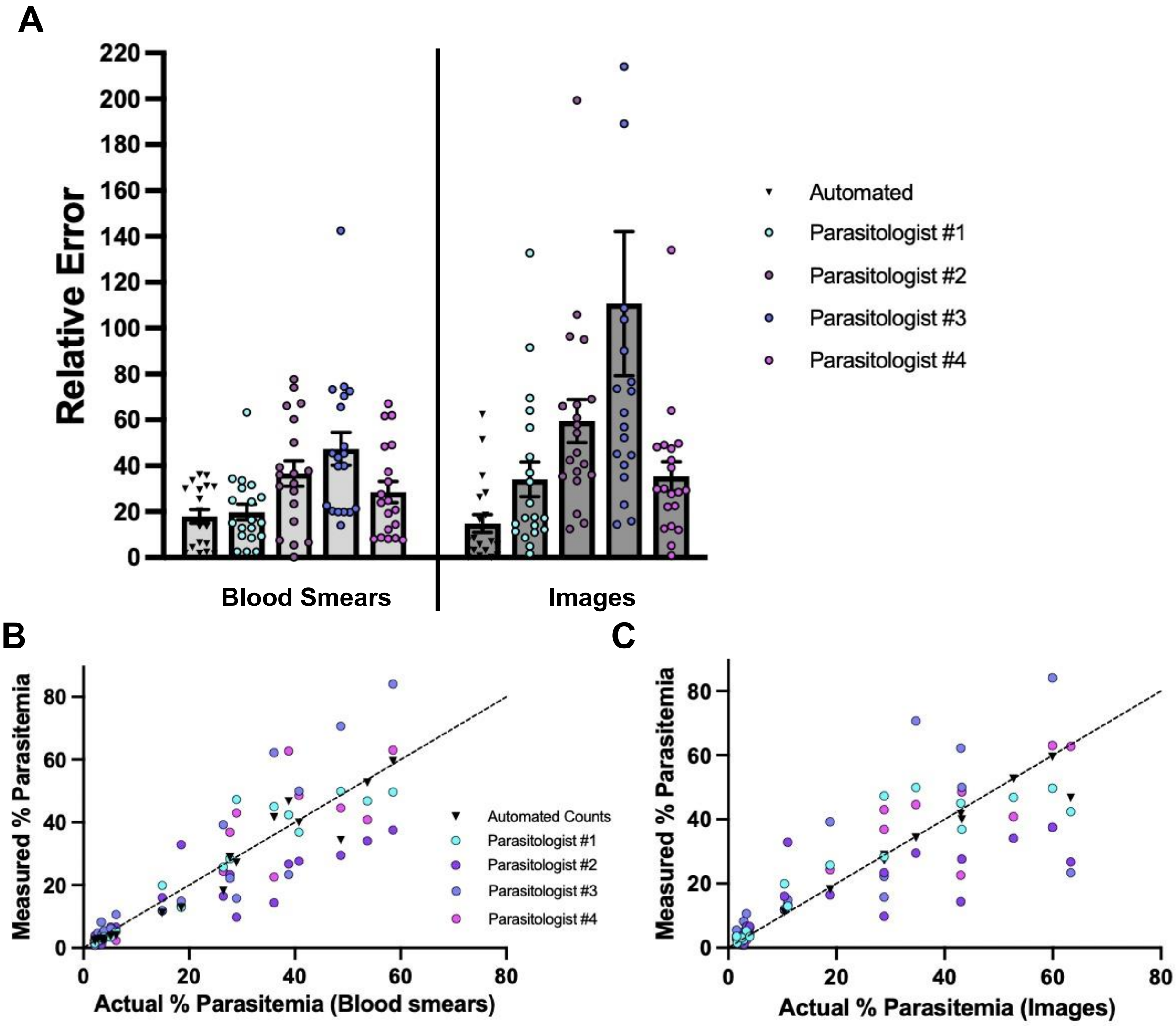

Supplementary Figure 4: Relative Error of Automated Method vs. Individual Parasitologists. A) Relative error values are shown for a single user determining % parasitemia by capturing images of blood smears and analyzing them using the automated model, Model_Rodent_RBCs (Black triangles), or four parasitologists manually counting infected/uninfected RBCs on the same blood smears under a

microscope (Par. #1 = Cyan, Par. #2 = Purple, Par. #3 = Blue, and Par. #4 = Magenta). Relative error is calculated for each blood smear as the absolute value of ((% parasitemia user – % *parasitemia reference standard*)/(% *parasitemia reference standard*)*100). *% parasitemia reference standard* was calculated using two different methods, by taking the mean % parasitemia of manual counts of the blood smears by 4 expert parasitologists (left) or by taking the % parasitemia counted by a single parasitologist counting every cell on images captured from the corresponding blood smears (right). B-C) % Parasitemia (y-axis) measured by capturing images of blood smears and analyzing them using the automated model (Model_Rodent_RBCs) (Black triangles) or four parasitologists manually counting infected/uninfected RBCs on the same blood smears under a microscope (Par. #1 = Cyan, Par. #2 = Purple, Par. #3 = Blue, and Par. #4 = Magenta). X-axis corresponds to the % parasitemia calculated using (B) the average of manual estimates of blood smears by all 4 parasitologists or from (C) a single parasitologist individually counting each cell in the images taken of the same blood smears.

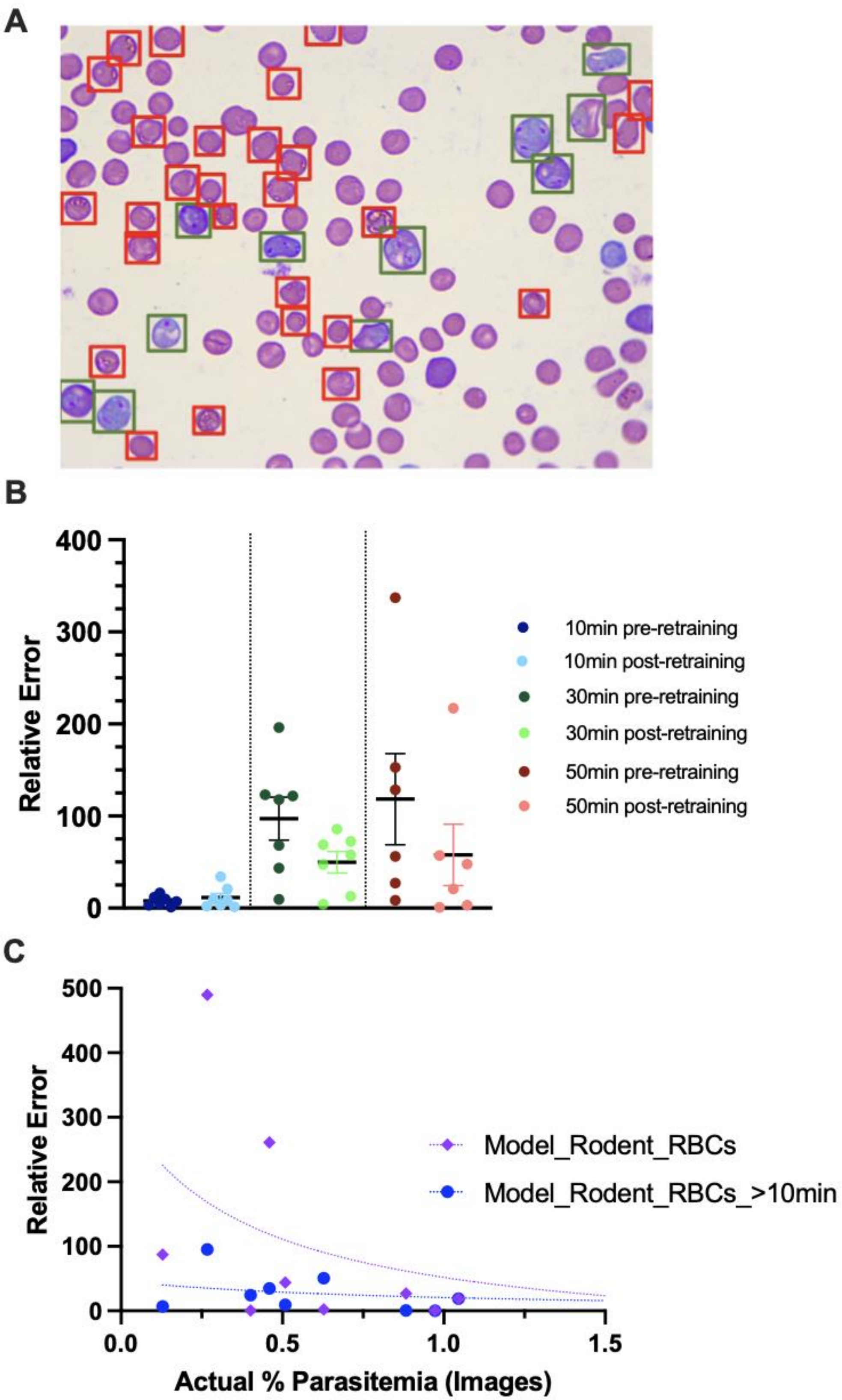
A
B
400
300
200
100
0
Relative Error
10min pre-retraining
10min post-retraining
30min pre-retraining
30min post-retraining
50min pre-retraining
50min post-retraining
C
500
400
300
200
100
0
Relative Error
Model_Rodent_RBCs
Model_Rodent_RBCs_>10min
0.0
0.5
1.0
1.5
Actual % Parasitemia (Images)

Supplementary Figure 5: Effect of Giemsa stain time on model performance. A) Example image is shown of a blood smear from a *P. berghei* infected mouse stained with Giemsa for 70 minutes. All RBCs annotated as infected by the original model (Model_Rodent_RBCs) are boxed. RBCs labeled as uninfected by the model are left unboxed. Green boxes represent infected RBCs that are true positives, and red boxes represent infected RBCs that are false positives. B) Relative error values are shown comparing detections by Model_Rodent_RBCs vs. manual counts using images from blood smears stained for 10min (dark blue, left), 30min (dark green, center), and 50min (dark red, right). Relative error is calculated for each mouse at one time point as the absolute value of ((% parasitemia automated count – % parasitemia manual count)/(% parasitemia manual count)*100). The model was then retrained with additional images from blood smears stained for 20min or 70min. This updated model (Model_Rodent_RBCs_>10min) was used to annotate the same images. Relative error using this updated model is shown to the right of original data points in light blue, light green, and light red respectively. C) Relative error of automated parasitemia measurements of *P. yoelii*-infected mice is shown from 0-6% parasitemia. Purple and blue dots represent relative error between automated and manual measurements for 1 mouse at 1 time point for the original and retrained model respectively. Relative error is calculated as the absolute value of ((% parasitemia automated count – % parasitemia manual count)/(% parasitemia manual count)*100). Purple and blue dotted lines represent nonlinear fit of relative error vs. % parasitemia for the original and retrained models respectively.

Supplementary Table 1. Training Configurations and Hyperparameters Used for Retraining (https://docs.ultralytics.com/modes/train/#train-settings)

Argument	Value	Description
optimizer	SGD	Choice of optimizer for training. Affects convergence speed and stability.
epochs	100	Total number of training epochs. Each epoch represents a full pass over the entire dataset. Adjusting this value can affect training duration and model performance.
batch	6	Batch size.
imgsz	1024	Target image size for training. All images are resized to this dimension before being fed into the model. Affects model accuracy and computational complexity.
lr0	0.00285	Initial learning rate (i.e. SGD=1E-2, Adam=1E-3) . Adjusting this value is crucial for the optimization process, influencing how rapidly model weights are updated.
lrf	0.396	Final learning rate as a fraction of the initial rate = (lr0 * lrf), used in conjunction with schedulers to adjust the learning rate over time.
momentum	0.98	Momentum factor for SGD or beta1 for Adam optimizers, influencing the incorporation of past gradients in the current update.
weight_decay	0.00054	L2 regularization term, penalizing large weights to prevent overfitting.
warmup_epochs	3.09	Number of epochs for learning rate warmup, gradually increasing the learning rate from a low value to the initial learning rate to stabilize training early on.
warmup_momentum	0.826	Initial momentum for warmup phase, gradually adjusting to the set momentum over the warmup period.
warmup_bias_lr	0.0308	Learning rate for bias parameters during the warmup phase, helping stabilize model training in the initial epochs.
box	0.0515	Weight of the box loss component in the loss function, influencing how much emphasis is placed on accurately predicting bounding box coordinates.
cls	0.966	Weight of the classification loss in the total loss function, affecting the importance of correct class prediction relative to other components.
cls_pw	0.908	cls BCELoss positive_weight
obj	0.2	obj loss gain (scale with pixels)
obj_pw	1.22	obj BCELoss positive_weight
Iou_t	0.2	IoU training threshold
anchor_t	3.9	Anchor-multiple threshold

anchors	2.0	Anchors per output layer
fl_gamma	0.0	focal loss gamma
hsv_h	0.0108	Adjusts the hue of the image by a fraction of the color wheel, introducing color variability. Helps the model generalize across different lighting conditions.
hsv_s	0.288	Alters the saturation of the image by a fraction, affecting the intensity of colors. Useful for simulating different environmental conditions.
hsv_v	0.167	Modifies the value (brightness) of the image by a fraction, helping the model to perform well under various lighting conditions.
degrees	0.114	Rotates the image randomly within the specified degree range, improving the model's ability to recognize objects at various orientations.
translate	0.0461	Translates the image horizontally and vertically by a fraction of the image size, aiding in learning to detect partially visible objects.
scale	0.219	Scales the image by a gain factor, simulating objects at different distances from the camera.
shear	0.0218	Shears the image by a specified degree, mimicking the effect of objects being viewed from different angles.
perspective	0.0	Applies a random perspective transformation to the image, enhancing the model's ability to understand objects in 3D space.
flipud	0.315	Flips the image upside down with the specified probability, increasing the data variability without affecting the object's characteristics.
fliplr	0.6	Flips the image left to right with the specified probability, useful for learning symmetrical objects and increasing dataset diversity.
mosaic	0.427	Combines four training images into one, simulating different scene compositions and object interactions. Highly effective for complex scene understanding.
mixup	0.0	Blends two images and their labels, creating a composite image. Enhances the model's ability to generalize by introducing label noise and visual variability.
copy_paste	0.0	Copies objects from one image and pastes them onto another, useful for increasing object instances and learning object occlusion.
